# Experience, Training Preferences, and Fighting Style Are Differentially Related to Measures of Body Composition, Strength, and Power in Male Brazilian Jiu Jitsu Athletes—A Pilot Study

**DOI:** 10.3390/sports11010013

**Published:** 2023-01-05

**Authors:** Christian G. Almeda, Gerald T. Mangine, Zackary H. Green, Yuri Feito, Duncan N. French

**Affiliations:** 1 Exercise Science, Kennesaw State University, Kennesaw, GA 30144, USA; 2 American College of Sports Medicine, Indianapolis, IN 46202, USA; 3 Ultimate Fighting Championship Performance Institute, Las Vegas, NV 89118, USA; 4 Health Sciences, Australian Catholic University, Melbourne 3065, Australia; 5 Medical and Health Science, Edith Cowen University, Perth 6027, Australia

**Keywords:** combat sports, muscular performance, grappling, martial arts, barbell velocity

## Abstract

To examine relationships between Brazilian Jiu Jitsu (BJJ) descriptors (belt rank, experience, gi preference, and fighting style), resistance training (RT) experience, and measures of body composition, strength (maximal handgrip, 3-5-repetition maximum [RM] in barbell glute bridge [GB], prone bench row [PBR], and bench press [BP]), and velocity (GB, PBR, and BP at 7 kg and 30–60% 1-RM), 13 experienced (4.3 ± 3.4 years) BJJ athletes were recruited for this cross-sectional, pilot study. Significant (*p* < 0.05) Kendall’s tau and Bayesian relationships were seen between belt rank and body fat percentage (τ = −0.53, BF_10_ = 6.5), BJJ experience and body fat percentage (τ = −0.44 to −0.66, BF_10_ = 2.6–30.8) and GB velocity (τ = −0.45 to −0.46, BF_10_ = 2.8–3.1), RT experience and strength (τ = 0.44 to 0.73, BF_10_ = 2.6–75.1) and velocity (τ = −0.44 to 0.47, BF_10_ = 2.6–3.3), gi preference-training and relative PBR strength (τ = 0.70, BF_10_ = 51.9), gi preference-competition and height and lean mass (τ = −0.57 to 0.67, BF_10_ = 5.3–12.4) and BP velocity (τ = −0.52 to 0.67, BF_10_ = 3.5–14.0). The relevance of body composition and performance measures to sport-specific training and research interpretation are differentially affected by a BJJ athlete’s experience (BJJ, belt rank, RT), gi preferences, and fighting style.

## 1. Introduction

Brazilian Jiu Jitsu (BJJ) is a grappling-based martial art that focuses on neutralizing an opponent [1,2,3]. Matches begin with both athletes standing but quickly progress to ground combat [4] where fighters attempt to score points or cause their opponent to submit from various strangulation (i.e., chokes), joint locks, or pressure techniques [3]. Athletes are generally active throughout an entire 5–10 min contest, alternating between low intensity efforts lasting 27–33 s, pauses (5–44 s), and shorter high-intensity efforts lasting 2–4 s [1]. Though athletes spend the majority of the match fighting for grip and holding opponents to set up a move, the higher intensity efforts may be considered “decisive” because their successful execution often allows an athlete to advance or preserve an important position (e.g., takedown, guard pass, mount or back mount, side control, knee on belly) [1,5,6]. A thorough understanding of the factors that contribute to successful execution of these moves might allow coaches and athletes to develop effective training regimen and competition strategies.

Beyond expected “technical skills”, fitness and flexibility, BJJ success is thought to be impacted by an athlete’s anthropometric characteristics and strength-power profile [2]. Most BJJ tournaments clearly acknowledge the influence of body mass on fighting ability by categorizing athletes according to weight class [3]. However, less is known about body composition. Comparative studies have either reported no differences in body fat percentage among athletes of different levels of skill and experience [7,8,9], differences based on fighting style [10], or lower body fat percentage in elite athletes [11]. The lack of consensus might be explained by inconsistent methods of estimating body fat percentage across studies, inconsistent study controls (e.g., whether the athlete is rapidly cutting weight leading up to a competition), the lack of comparisons between body compartments, and possibly the modulating effect of weight class (i.e., body composition may be differentially relevant across weight classes). Still, a more favorable ratio between functional (e.g., skeletal muscle) and non-functional (e.g., adipose tissue) mass might allow a fighter to better sustain effort [12,13] and having more lean mass generally translates to greater strength and power [14,15,16]. Indeed, isometric [9,17,18] and dynamic [11,17] assessments have been used to demonstrate greater strength in higher ranking or more experienced BJJ athletes [9,11], but not always [11,17,18]. Andreato and colleagues [2] proposed that the relevance of isometric grip strength might be affected by the stimulus’ angle (i.e., grip angle while fighting) and whether an athlete trains or competes with a kimono or gi (i.e., the traditional BJJ uniform). The collar, sleeves, belt, and pants of the traditional BJJ uniform provides more “gripping” opportunities compared to no-gi competitions, which are typically fought in tight-fitting compression clothing [3]. The tighter clothing used in no-gi competitions necessitates the use of more wrestling-style grips and a greater reliance on hip positioning. The athlete’s preference and training habits on this matter might influence the importance they place on developing grip strength, but this has been left mostly unexplored. Likewise, resistance training experience is also well known to affect maximal testing performance [19] but without its consideration, any observed differences among athletes may simply be coincidental. Still, in grappling sports, stronger fighters are considered to have an advantage when combat progresses to the ground (e.g., performing and escaping immobilization techniques) [20,21].

Successful execution of decisive moves, where the fighter attempts to advance on or defend against a resisting opponent, requires precise expression of strength and speed (i.e., power) [2,20,21,22]. Although this need is acknowledged, few have studied high-velocity movements in BJJ athletes and most have limited their analysis to jump performance [2]. Jumping performance is an accepted proxy for describing lower-body power and has been shown to distinguish between novice and expert BJJ athletes [9]. However, the majority of a BJJ match is spent in ground combat [4] and jumping may not adequately represent the qualities a fighter needs to execute decisive techniques. With this in mind, recent attention has been given to the importance of (and lack of research about) horizontal kinetic expression to sports performance [23,24], which might be assessed via a barbell glute bridge (or hip thrust). Like more commonly recommended traditional resistance training (e.g., squat, deadlift) and weightlifting techniques (e.g., clean, snatch), the glute bridge and hip thrust exercises train musculature relevant to ground combat but in a more sport-specific direction [21,23]. Thus far, and to the best of our knowledge, no study has examined this exercise in BJJ athletes. In fact, very limited attention at all has been placed on characterizing kinetics in exercises besides a vertical jump in BJJ athletes [2]. Da Silva and colleagues [22] examined bench press power at 30–60% of maximal strength but found no differences between more and less experienced BJJ athletes. Rather, their main finding was that peak power expression occurred when using a load approximately equal to 42% of maximal strength. A later study reported that peak power expression occurred at a similar relative intensity load (45–50%) for the prone bench row [25], but made no comparisons between athletes or related these efforts to BJJ performance.

Successful belt progression prioritizes the development of technical and strategic skills through practice and study of the sport [26]. Accordingly, BJJ tournaments typically divide competitors by belt rank to facilitate fair competition between fighters of similar skill and expertise. The assumption is that a higher-ranking athlete will have been exposed to a greater number of techniques and had more time to master learned skills and strategies that could provide a competitive advantage [27,28,29]. However, a fighter’s superiority in one or more relevant physiological traits [1,2,30] could modify whether an advantage still exists between similarly skilled opponents, and aptitude in such traits may not occur comcomittantly with belt progression. Belt progression from white to black may take between 5 and 10 years, with 6 months to 3 years separating individual belt ranks, depending on a BJJ academy’s specific federation affiliation and the criteria set forth by the academy instructor [26]. Meanwhile, a number of factors (e.g., age, sex, training status, prescription) differentially affect the developmental timeline for the various physiological characteristics relevant to BJJ [19,21,31,32]. Thus, there may be a disconnect between belt rank, BJJ training experience, and the combative skill of the athlete. Conversely, superiority observed in any measure derived from a resistance training exercise may simply reflect the athlete’s experience and familiarity with that modality. The question of whether these descriptive factors are differentially related to the measures often cited as being relevant to the sport has not been answered.

The purpose of this pilot study was to begin examining differences in relationships between belt rank (a proxy of ‘sporting expertise’) and metrics of training experience (BJJ and resistance training), and the physiological measures often assessed in BJJ athletes (i.e., body composition, strength, and power). A secondary aim was to examine the influence of gi preference (during training and competition) and fighting style (i.e., defensive, guard/pulling guard or offensive, take down/pass the guard) on these measures. Regarding body composition, it may be hypothesized that higher ranking athletes and those with more training experience (BJJ and/or resistance training) would also possess a more ideal ratio between lean (greater) and fat mass (less) due to the assumption that these descriptors would reflect longer periods of being physically active. However, body composition may also be modified by fighting style where defensive fighters can be expected to possess greater lean and fat mass [10]. Strength and power are often assessed via traditional resistance training exercises and athletes with greater resistance training experience are likely to be more familiar with these movements and outperform those with less experience. In contrast, because greater emphasis is placed on technical skill advancement [26] BJJ belt rank and experience are not expected to be related to most measures of strength and power. An exception is possible with hand grip, where fighters who rely on grip during training and competition (i.e., those who prefer a gi) may also possess greater grip strength. The findings of this study will be useful for helping coaches and athletes determine the most relevant training targets, and to help guide future studies on the phyisiological predictors of BJJ performance.

## 2. Materials and Methods

### 2.1. Experimental Design

Recreationally trained men in both BJJ and resistance training were recruited for this study via word of mouth, social media, and flyers posted at training facilities located throughout the local metropolitan area. Participation required two visits to the exercise physiology laboratory, and each visit required athletes to abstain from vigorous exercise for at least 48-h, avoid alcohol consumption for 24-h, and maintain their normal hydration and dietary habits throughout the 1-week study. This included their normal supplementation and caffeine intake habits, all of which were verified via 24 h food recalls completed on each visit. Athletes were also asked to arrive fasted for 8 h on the first visit to complete body composition assessments. Afterwards, athletes were then given the opportunity to consume a light snack (granola bar, fruit, etc.) and were then familiarized with all strength and performance testing procedures. The familiarization protocol provided athletes an opportunity to practice and verify all technical standards for a maximal isometric handgrip strength (MIHS) test, the barbell glute bridge (GB), prone bench pull (PBP), and bench press (BP). The first visit concluded maximal strength assessments. Athletes returned to the Exercise Physiology Laboratory within 2–7 days to complete all velocity-based performance tests. The University’s Institutional Review Board approved all testing procedures and protocols (IRB #19-444).

### 2.2. Participants

A convenience sample of thirteen men (26.5 ± 5.1 years, [20–36 years]; 176.5 ± 9.2 cm [158.1–194.3 cm]; 78.7 ± 12.7 kg [58.6–96.7 kg]) with BJJ (4.3 ± 3.4 years [0.5–13 years]) and resistance training (7.0 ± 3.7 years [0.5–14 years]) experience volunteered for this study. To be consistent with previous studies, individuals from the ultra-heavyweight division (>100 kg) were excluded from this investigation [6,33]. At the time of enrollment, all participants were required to have been training with both modalities on at least two sessions per week. The sample included white belts (n = 4), blue belts (n = 3), purple belts (n = 5), and a black belt (n = 1). Athletes reported practicing BJJ on 3.9 ± 1.4 days per week and completing 1.6 ± 0.7 sessions per day. Eight athletes reported preferring to practice with a gi, while 2 preferred practicing without a gi and 3 had no preference. All but 2 athletes had reported having competed in 1.7 ± 0.8 BJJ tournaments per year, with 4 having competed in a tournament within the 6 months prior to this study. Additionally, athletes possessed 7.0 ± 3.7 years of resistance training experience. Seven athletes reported training with the BP exercise, six incorporating grip strength specific exercises, and three utilizing the PBP exercise. All athletes were free of any cardiovascular, metabolic, or renal disease, as well as any musculoskeletal impairments that could affect performance (determined a health and physical activity history questionnaire), and each provided his written informed consent to participate.

### 2.3. Training and Competition Preferences

During enrollment, all athletes were asked questions about their training experience and preferences, as well as their fighting style preferences (via health and physical activity history questionnaire). Athletes were asked to indicate their current belt rank and these were codified for analysis using the following values: 1 = white; 2 = blue; 3 = purple; 4 = brown; 5 = black. Likewise, athletes were asked about their gi preference during training (1 = gi; 2 = no preference; 3 = no gi), and during competition (1 = gi; 2 = equal; 3 = no gi). For athletes who had not participated in a BJJ competition, no value was given (i.e., the cell was left blank) because any value would have affected the statistical analysis. Athletes were also asked about their preferred fighting style. Those who preferred the guard/pulling guard were assigned “1”, whereas those who preferred to take down/pass the guard were assigned “3”; those who had no preference were assigned “2” for statistical analysis.

### 2.4. Body Composition Assessments

Initially, height (±0.1 cm) and weight (±0.1 kg) were assessed using an electronic physician’s scale (Tanita WB 3000, Arlington Heights, IL, USA) with the athletes standing barefoot, with feet together, in their normal daily attire. Subsequently, body composition was assessed using dual energy X-ray absorptiometry (DXA; General Electric Lunar iDXA, Boston, MA, USA). Briefly, athletes removed any metal or jewelry and laid supine on the scanning table. An entire body scan in “standard” mode was used to estimate body fat percentage (BF%), total fat mass (kg), total and regional non-bone lean mass (NBLM, kg), bone mineral density (BMD, g⸱cm^−2^), and total and regional bone mineral content (BMC, kg) using the company’s recommended procedures and supplied algorithms. Quality assurance was assessed by daily calibrations performed prior to all scans using a calibration block provided by the manufacturer. All iDXA measurements were performed by the same researcher using standardized subject positioning procedures. Obtaining total and regional estimates via DXA had been previously reported to be reliable (ICC’s > 0.94) in 10 healthy, recreationally active adults (25.1 ± 2.4 years; 81.1 ± 18.5 kg; 175.7 ± 6.8 cm) [34]. The body composition characteristics of the present study’s sample are presented in Table 1.

### 2.5. Strength Assessments

Strength assessments were completed on the first visit and began with MIHS testing. Since the MIHS assesses strength in small muscle groups, it did not require an extensive warm-up protocol [31]. After being familiarized with testing procedures, athletes immediately progressed to three submaximal warm-up sets at 25%, 50%, and 75% of their perceived maximal effort before completing three maximal trials on each hand (a total of six maximal trials) beginning with their self-selected dominant (i.e., preferred writing, throwing) hand. Each maximal trial was separated by 2 min of rest and athletes alternated hands between maximal attempts [5,31]. All maximal trials were performed using a handgrip dynamometer (Jamar Plus+, Greendale, WI, USA) and athletes were instructed to squeeze the dynamometer as forcefully as possible for 3 s while holding it alongside their body at 90° [31,35]. The highest value from 3 trials (±0.1 kg) was retained for the dominant (identified by athlete) and non-dominant hands [36]. Additionally, total grip strength (dominant + non-dominant) and bilateral strength asymmetry ([stronger hand–weaker hand]/stronger hand × 100) [37] were calculated for statistical analysis.

Athletes then progressed to a standardized warm-up for strength assessment of the GB, PBR, and BP. The standardized warm-up began with five minutes of cycling on a stationary bike at a self-selected pace before progressing to a series of dynamic exercises made specific to each tested movement. That is, a series of dynamic stretches and then three sub-maximal warm-up sets were completed immediately before each tested movement. Athletes performed 10 repetitions of each dynamic exercise prescribed for the GB (walking toe touches, air squats, walking lunges, and bodyweight glute bridges), PBR (arm circles, banded face pulls, and barbell bent over rows) and BP (arm circles, arm swings, and push-ups). Then, they completed one set of 8 repetitions at 25% of their estimated one-repetition maximum (1-RM), a second set of 5 repetitions at 50% of their estimated 1-RM, and then a final warm-up set of 3 repetitions at 75% of estimated 1-RM. Each athlete was then allowed up to 3 maximal trials to find his 3–5 repetition-maximum, which was then used to estimate their 1-RM for each exercise [32]. Athletes were given 2–3 min between and after warm-up sets and 3−5 min of rest between maximal attempts. All warm-ups and subsequent maximal testing were completed using a standard Olympic barbell and bumber weights (Promaxima Manufacturing, Houston, TX, USA) under the supervision of a certified strength and conditioning specialist (CSCS).

**Figure 1:**
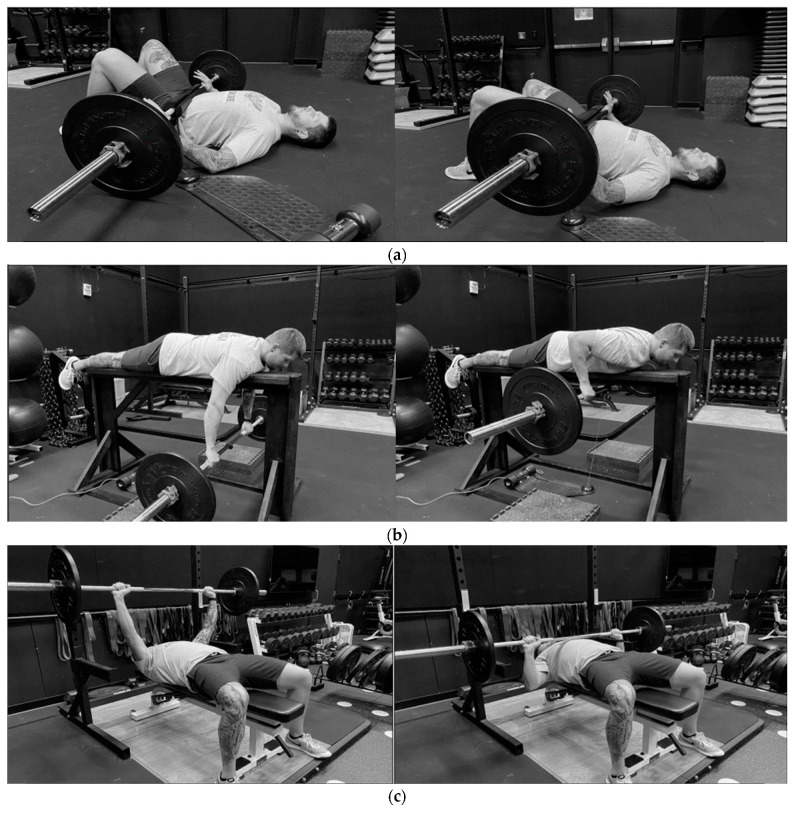
Starting and finishing positions for the (**a**) Glute Bridge, (**b**) Prone Bench Row, and (**c**) Bench Press.

Technical standards for each exercise were enforced by the CSCS and any attempt that failed to meet these standards was discarded. Athletes initiated the GB with their feet (approximately shoulder-width apart), upper back, buttocks, and head placed firmly on the testing surface, and knees bent at approximately 90°. The athletes used their hands to support a loaded barbell placed across their hips. On their ready, athletes were instructed to lift the barbell vertically by raising their hips to full extension before returning to the starting position under control and repeating for 3–5 repetitions. Repetitions were discarded if the athlete failed to maintain contact between the testing surface and their head, upper back, and feet. For the PBR, athletes were laid prone on an elevated bench that placed them approximately four feet from the ground. They were instructed to grasp a loaded barbell located directly beneath them on the ground and hold it just above the ground with their arms at full extension. Researchers assisted the athlete in lifting the barbell to the starting position when it too far out of their reach. On their ready, athletes pulled the barbell vertically towards their body until it touched the bottom of the bench before lowering it to the starting position under control. Repetitions were discarded if the athlete’s torso did not maintain contact with the bench. For the BP, athletes were required to maintain contact between their feet and the floor, and between the bench and their buttocks, shoulders, and head. All repetitions began with the athlete holding the loaded barbell over their chest, arms extended, and hands slightly wider than shoulder-width. Athletes then lowered the bar to their chest under control and immediately pressed it back to the starting position. Repetitions that involved excessive bouncing of the bar, arching of the back, or failure to maintain 5-point contact were discarded. All BP trials were performed with the CSCS as a spotter and within a standard weightlifting rack with safety bars set at a height that would prevent an uncontrolled barbell from making full contact with the torso, neck, or head. The start and finishing positions for each exercise are portrayed in Figure 1, while strength characteristics are presented in Table 1.

### 2.6. Velocity-Based Performance Assessments

**Figure 2:**
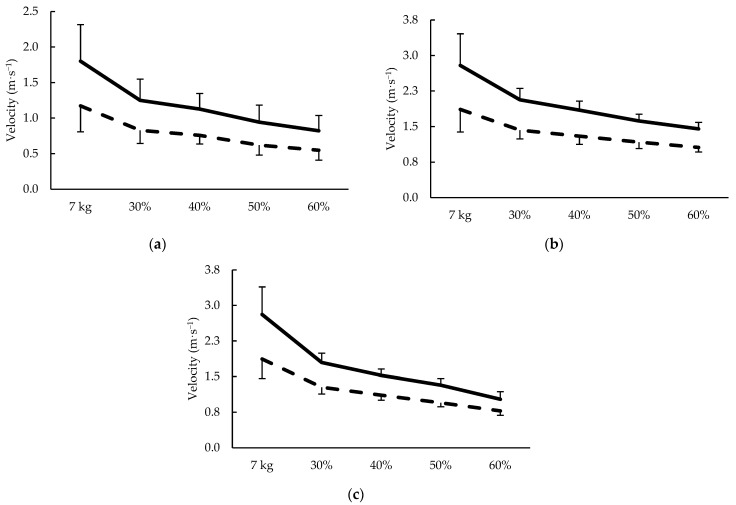
Peak (solid line) and average (dashed line) barbell velocity during the (**a**) glute bridge, (**b**) prone bench row, and (**c**) bench press exercises at sub-maximal loads.

Velocity-based assessments for the GB, PBR, and BP were completed on the second visit. Prior to testing, athletes completed the same standardized warm-up described for the first visit. Following the warm-up, athletes were asked to complete on set of 3 repetitions at 7 kg to simulate an “unloaded” movement (i.e., <5% of body mass and <10% of 1-RM in each lift), and then 30%, 40%, 50%, and 60% of the 1RM obtained on visit 1 for each exercise. These percentages have been previously used to examine barbell velocity within this population [22,25]. Barbell velocity was monitored during each repetition using a linear position transducer (Tendo Weightlifting Analyzer, TENDO Sports Machines, Trencin, Slovak Republic). The transducer was placed on the ground directly beneath the barbell’s starting position and connected to the barbell via an extended cable. The positioning of the transducer was such that the extended cable’s angle was approximately 90° to the ground, which helped to minimize its horizontal displacement during each repetition. Data from every repetition was collected via the TENDO Unit Computer Software Version PA (v6.06, TENDO Sports Machines, Trencin, Slovak Republic) and it provided estimates of peak (V_PK_; ±0.01 m⸱s^−1^) and average (V_AVG_; ±0.01 m⸱s^−1^) velocity. The average V_PK_ and V_AVG_ across all 3 repetitions at each load was calculated and retained for statistical analysis. The TENDO has previously been reported to be reliable for measuring barbell velocity across multiple resistance training lifts and intensities (ICC’s > 0.91) in 10 active, resistance-trained men (26.8 ± 3.5 years; 92.6 ± 6.5 kg; 180.5 ± 6.6 cm) [38]. Barbell velocity expression at each sub-maximal load for each exercise is illustrated in Figure 2.

### 2.7. Statistical Analysis

Relationships between belt rank, training experience (BJJ and resistance training), gi preference (in training and competition) and physiological measures of body composition, strength, and power were analyzed by using both a frequentist and Bayesian approach. Since several variables of interest were ordinal by nature, relationships were examined by calculating Kendall’s tau (τ) correlation coefficients. The strength of observed relationships were interpreted using the following criteria: Trivial (<0.10), small (0.10–0.29), moderate (0.30–0.49), high (0.50–0.69), very high (0.70–0.90), or practically perfect (>0.90) [39]. Meanwhile, the Bayesian approach assessed the likelihood of observed relationships under the alternative hypothesis compared to the null hypothesis (i.e., no relationships between variables) by calculating Bayes factors (i.e., BF_10_) for each comparison using default prior scales [40]. These were interpreted according to the recommendations of Wagenmakers et al. [41] where a correlation was interpreted as evidence in favor of the null hypothesis when BF_10_ < 1. Otherwise, evidence in favor of the alternative hypothesis was interpreted as “anecdotal” (1 < BF_10_ < 3), “moderate” (3 < BF_10_ < 10), “strong” (10 < BF_10_ < 30), “very strong” (30 < BF_10_ < 100), or “extreme” (BF_10_ > 100). All statistical analyses were performed using JASP 0.16.1 (Amsterdam, the Netherlands) with a criterion alpha set at *p* ≤ 0.05. All data are reported as mean ± standard deviation.

## 3. Results

### 3.1. Body Composition

**Figure 3:**
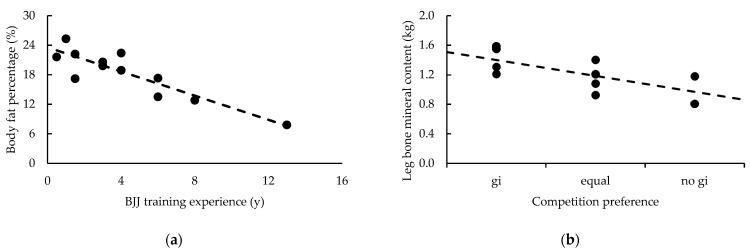
*Strong* relationships between (**a**) BJJ experience and body fat percentage, and (**b**) bone mineral content of the legs and gi preference in competition.

Except for belt rank and BF% (*p* = 0.020), and BJJ experience and BF% (*p* = 0.002) and fat mass (*p* = 0.041), evidence was either *anecdotal* or in favor of measures of body composition not being related to belt rank or training experience (BJJ and resistance training). Likewise, gi preference during training was not related to any measure of body composition. However, *moderate* evidence suggested negative relationships between gi preference during competition and the athletes’ height (*p* = 0.027), trunk NBLM (*p* = 0.042), trunk BMC (*p* = 0.027), and total BMC (*p* = 0.027). Negative relationships in this context imply that a preference towards using a gi in competition is associated with being taller or possessing more NBLM and BMC. *Strong* evidence also suggested a negative relationship between leg BMC and gi preference in competition (*p* = 0.011). Relationships with measures of body composition are presented in Table 2, while the *strong* relationships observed between BJJ experience and BF%, and between leg BMC and gi preference in competition are illustrated in Figure 3.

### 3.2. Maximal Strength

**Figure 4:**
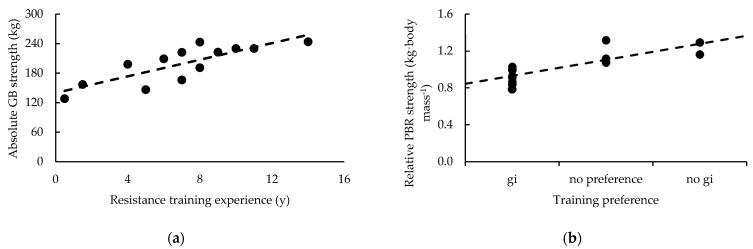
*Very strong* relationships between measures of strength and (**a**) resistance training experience and (**b**) gi preference during training.

Evidence favored the null hypothesis, that belt rank, training experience (BJJ and resistance training), gi preference (training and competition), and fighting style were not related to any MIHS measure. Likewise, evidence favored no relationships between BJJ experience (belt rank, years of experience) and 1-RM strength (absolute and relative) in the GB, PBR, and BP exercises. Evidence was also either *anecdotal* or in favor of 1-RM strength measures not being related to gi preference (training and competition), except for *very strong* evidence suggesting a positive relationship between gi preference during training and relative PBR strength (*p* = 0.003); implying that those with greater relative strength preferred not to use a gi during training. Meanwhile, except for PBR relative strength, evidence ranged from *anecdotal* to *very strong* for positive relationships between resistance training experience and all measures of 1-RM strength (absolute and relative); the strongest being absolute GB strength (*p* < 0.001). Relationships with measures of strength are presented in Table 3, while the *very strong* relationships observed between measures of strength, resistance training experience, and gi preference during training are illustrated in Figure 4.

### 3.3. Velocity-Based Performance Assessments

**Figure 5:**
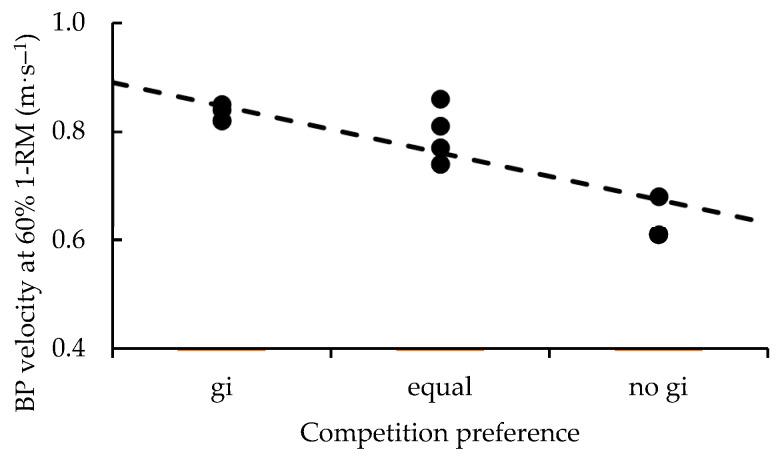
*Strong* relationship between gi preference in competition and BP barbell velocity at 60% 1-RM.

Evidence was also either *anecdotal* or in favor of belt rank, gi preference during training, and fighting style not being related to barbell velocity in any exercise at any load. *Anecdotal* to *moderate* evidence favored negative relationships between BJJ experience and peak GB barbell velocity expressed at 60% 1-RM (*p* = 0.040) and average GB barbell velocity expressed at 50–60% 1-RM (*p* = 0.035). *Anecdotal* to *moderate* evidence favored a negative relationship between resistance training experience and peak BP barbell velocity expressed at 40% 1-RM (*p* = 0.037) and positive relationships to average GB barbell velocity expressed using 7 kg (*p* = 0.037) and peak PBR barbell velocity expressed at 60% 1-RM (*p* = 0.027). *Moderate* to *strong* evidence favored negative relationships between gi preference in competition and average BP barbell velocity expressed at 30% (*p* = 0.048), 50% (*p* = 0.026), and 60% 1-RM (*p* = 0.010). Relationships with measures of barbell velocity are presented in Table 4, while the *strong* relationship between gi preference in competition and BP barbell velocity at 60% 1-RM is illustrated in Figure 5.

## 4. Discussion

This study examined the influence of BJJ athletes’ training background and training-competition preferences (fighting style and gi) on measures of body composition, strength, and power. Athletes were classified by belt rank, training experience (BJJ and resistance training), gi preference (during training and competition), and fighting style. Total and regional estimates of body fat, NBLM, BMC were then collected before athletes’ maximal handgrip strength, and strength and power in GB, PBR, and BP were assessed. Relationship analysis suggested that higher ranking (i.e., belt rank) BJJ athletes possessed lower body fat percentages, but a stronger relationship was seen with BJJ experience. To a lesser extent, more experienced BJJ athletes possessed less fat mass and interestingly, performed high-load GB (50–60% 1-RM) at slower velocities. In contrast, athletes with more resistance training experience were stronger in each lift and performed GB and PBR at greater velocities with select loads. Meanwhile, body composition and strength also appeared to be distinguished by gi preference. Athletes who preferred training without a gi possessed greater relative PBR strength, while those who competed with a gi were taller, possessed more trunk NBLM and more BMC, particularly in the legs and trunk. No other significant relationships were observed in this group of recreationally trained athletes. Although the influence of body composition, strength, and power measures have been previously documented in BJJ athletes [1,2,30], this appears to be the first study to examine how experience and training habits influence these measures and distinguish belt rank, years of BJJ training experience, and years of resistance training experience.

Higher ranking (i.e., belt rank) BJJ athletes possessed lower body fat percentages, but a stronger relationship was seen with BJJ experience. Previous studies examining the role of body composition on BJJ performance lack agreement [7,8,9,10,11]. Marinho and colleagues [11] observed lower body fat percentages among black and brown belt BJJ medalists in national and/or international competition compared to non-medalists. Meanwhile, a similar comparison was performed between black belts who either competed or did not compete in the 2010 World Championships [7], and no differences were found. No differences in body compositions have also been reported when the sample only included national/international competition medalists [8] and when groups were formed by arbitrary delineations based on years of experience (greater or less than 4 years) and belt rank (higher or lower than blue belt) [9]. The lack of agreement cannot be currently explained but may involve the several methodological differences that exist across each of these studies. Each study utilized a different method for defining groups, BJJ skill, and experience, and then used different methods for assessing body composition. One study did not report its method of body composition assessment [7] and two estimated it via skinfold thickness with one measuring 3 sites [11] and the other measuring 7 sites [8]. Though a historically common and accessible method, the validity and reliability of skinfold analysis is influenced by the number of sites assessed, based on several assumptions about body density, heavily dependent on the practitioner’s technique, and subject to interindividual variation [42]. Many of these limitations are overcome when using technology. For instance, Diaz-Lara et al. [9] used bioelectrical impedance analysis (BIA) to compare novice and expert BJJ athletes. However, BIA devices rely on nutritional and hydration status being well controlled and their accuracy is dependent on the sophistication of their programmed algorithms [42]. In that study, novice and expert BJJ athletes were enrolled and assessed on the day of a tournament (prior to their first match), making it highly unlikely (not reported in methodology) that pre-assessment criteria about nutrition and hydration status were met, while the specific device used in that study (BC-418. Tanita Corp, Japan) was discontinued within a year of the study’s publication. The present study attempted to overcome these limitations by verifying participant consistency with factors that would impact hydration status and using a more advanced and comprehensive technology. Our findings support the idea that body composition and BJJ skill are related, regardless of whether skill was defined by years of experience or belt rank. It might be hypothesized that experience and rank progression emphasizes the need to gain or maintain a healthy ratio of lean to fat mass to better sustain effort [12,13] or express force and power [14,15,16] during decisive moves. Future studies seeking to confirm or refute this hypothesis are encouraged to utilize consistent and standardized methods to facilitate generalized conclusions.

Athletes in this study were asked whether they preferred to ‘guard’ or ‘pass the guard’ during a match, but their preference was not related to their body composition, strength, or barbell velocity expression. This finding is not consistent with the findings of Báez et al. [10], who noted differences in somatotype traits in BJJ athletes who preferred one of these fighting styles over the other. In that study, pass fighters were shorter and exhibited greater mesomorphic traits than guard fighters who possessed more ectomorphic traits. Pass fighters attack more often and thus, might require greater strength, speed, and power to successfully execute their strategy. Mesomorphs are more muscular than other somatotypes and greater muscle mass positively affects force and power expression force [15,16]. Meanwhile, a taller ectomorph might better be able to create space and defend against a pass fighter’s attack. However, neither the present study nor that conducted by Báez et al. [10] could adequately confirm these suppositions. Báez et al. [10] estimated body composition characteristics from circumference and skinfold measurements and did not see any differences in muscle mass between fighting styles. It is possible that such differences were missed due to the sophistication of these assessment methods, as they rely on several assumptions and introduce multiple sources of error between their actual measurement and then being placed into several estimation equations [42]. Although our study used a more advanced method for obtaining body composition estimates, as well as strength and power, our participants were given the additional option of stating that they did not have a fighting style preference. That combined with a smaller sample size may have limited our statistical power to observe a relationship.

An important finding demonstrated by this study that should be accounted for in future investigations that intend on using traditional measures of strength and power to explain BJJ performance was the role of resistance training experience. Amongst all strength/power variables, only three instances were observed where BJJ experience was related to performance (peak GB velocity at 60% 1-RM, average GB velocity at 50–60% 1-RM), and these were negatively related. At this time, it cannot be explained why a more experienced BJJ athlete would perform the GB exercise more slowly at these specific loads. A recent study noted a lack of comcomitant horizontal jumping (i.e., broad jump) performance following 14-weeks of hip thrust training and related strength increases [24]. It is possible that more experienced athletes might find themselves in this position less, and even when they do, they rely more on their technical skill than their physical attributes. A kinematics-based study would be useful to confirm whether the velocity profile of specific fighting movements related to GB are technically optimized when performed in a more slow and controlled way. Alternatively, since the GB testing position is seen more often in guard fighters during a match, it is possible that fighting style could have impacted the observed relationships between BJJ experience and measures of strength and power. BJJ experience was different among the fighting styles examined in this study (guard = 4 years, pass guard = 2.8 years, and no preference = 5.6 years), but these values are representative of 2, 6, and 5 athletes, respectively. A larger and more evenly distributed sample would have been necessary to adequately assess the partial effects of a third variable on the examined relationships. Regardless, it appears to be anecdotally accepted, and strongly implied by common belt progression criteria [26], that strength and power may be of secondary importance compared to technical skill and fighting strategy. If so, any relationships between BJJ experience and measures of strength and power would be highly individualistic at best or simply non-existent among those who do not value these traits (i.e., selection bias). In contrast, athletes who historically placed more importance on these traits and thus, possessed greater resistance training experience, consistently performed better in most measures examined in this study.

Though athletes may prefer to guard or pass the guard in a match, doing so is based on opportunity and the actions of one’s opponent. Meanwhile, years of participation in BJJ, resistance training, or any sport/art does not necessarily equate to aptitude since the quality of those experiences may vary considerably across individuals. Athletes might attempt to place themselves in various situations that could expose them to certain types of experiences, but ultimately, how these experiences play out cannot be controlled. More control is accomplished when athletes clearly define the context of a fight by training or competing with or without a gi. This decision forces a change in strategy because it influences the availability of certain grips and associated techniques. The findings of this study indicate that gi preference was related to body composition and strength. Athletes who prefer to train without a gi also possessed greater relative strength in the PBR exercise, an exercise was meant to resemble the athlete’s ability to control an opponent’s center of mass by pulling them out of position. Interestingly, this did not coincide with other relevant pulling motion attributes (i.e., grip strength or barbell velocity). An athlete’s grip on the barbell is likely to be a limiting factor for PBR, while successfully moving one’s opponent requires a combination of strength, speed, and positioning. On one hand, no-gi fights present less “gripping” opportunities [3] and thus, it may be less necessary to grip an opponent when executing similar techniques (to those used in gi fighting). Alternatively, the isometric maximal handgrip strength test used in this study may have simply lacked specificity [2]. Likewise, it is unlikely that a fighter will execute a pulling move by simultaneously pulling their opponent with both arms in the fashion PBR was performed in this study. However, evidence supporting or refuting these ideas is non-existent. Aside from the present investigation, only one other study examined PBR in BJJ athletes and it aimed to identify the load in which peak power is expressed [25]. Future studies may better understand the role of these movements in no gi fighting by incorporating a variety of conditions (e.g., dynamic and isometric grip at different wrist/hand positions) when assessing grip.

Comparatively, the only velocity-based measure related to gi preference involved average barbell velocity during BP. Athletes who preferred to compete with a gi executed this movement with greater speed at 30%, 50%, and 60% 1-RM; there was *anecdotal* evidence for 40% as well. Previously, bench press power at these loading ranges was found to be similar amongst BJJ athletes of varying levels of experience [22]. Though our data supports that outcome, it also presents a potentially different lens through which BP velocity may be related to BJJ performance. Gi fighters in this study were also taller and possessed more NBLM (trunk) and BMC (legs and trunk). Along with there being no significant relationships to other velocity-based measures, it is possible that this specific combination of measures may be related to tactical strategies that are unique to gi fighting. Greater skeletal and NBLM mass imply a greater ability to produce force [14,15,16,43], while being tall and being able to explosively “punch” might have something to do with strategically creating space. However, this study’s sample was not large enough to examine the combination of gi preference and fighting style and how various combinations might relate to body composition, strength, and power.

## 5. Conclusions

Previous studies have attempted to determine the physiological relevance of various measures to BJJ amongst BJJ athletes of different levels of skill [7,8,9,10,11,22], but the findings have not been consistent. The lack of uniformity may have been caused by various methodological shortcomings, some of which we have attempted to overcome in this study. Instead of comparing formed groups based on arbitrary thresholds for defining experience and skill (e.g., belt rank above or below a specific color; success or failure at specific competitions), we viewed experience as a continuous variable and observed that relationships differed when defining skill by years of experience versus belt rank. Years of experience was negatively related to body fat percentage and, interestingly, GB barbell velocity, whereas no relationships were observed with belt rank. Previous studies have also failed to account for the role of resistance training experience on measures of strength and power. Here, we observed greater strength in nearly all measures of dynamic strength in athletes who were more experienced with resistance training, suggesting that the importance that individual athlete’s place on this training modality will affect associated performance outcomes and accompanying relationships to BJJ performance. Finally, we examined the roles of both fighting style and gi preference, and used a more comprehensive assessment of body composition (i.e., DXA) compared to somatotype, skinfold thickness, and BIA methods used by others. With these we we noted that gi preference was a better indicator of body composition (measured via DXA), strength, and power than fighting strategy itself. An unfortunate limitation to this study, however, was that our sample size was blunted because data collection initiated immediately prior to a global pandemic and conditions were too variable across athletes upon their return to continue the study. Future investigations are encouraged to expand our findings and include several of the important methodological considerations highlighted by this pilot study.

Outside of sport-specific practice, athletes and coaches will utilize a strength and conditioning regimen to develop physical and physiological characteristics known to impact performance, and periodically test their progress. The findings of this study suggest that several population characteristics among BJJ athletes will affect the results of such tests. How BJJ athlete’s skill and experience are defined (i.e., belt rank or years of experience), their familiarity with resistance training, their gi preference during training and competition, and fighting style were all found to impact relationships to measures of body composition and performance. Thus, the importance of training for different body composition characteristics, types of strength, and movement pattern velocity may vary across population subsets. Careful consideration should be given to the relative importance of each targeted characteristic when developing a sport-specific training regimen. Meanwhile, these characteristics should be accounted for in future investigations into the factors that impact BJJ performance.

## Figures and Tables

**Table 1:** Body composition and strength characteristics of Brazilian Jiu Jitsu athletes.

	Mean ± SD	Range
Body fat percentage (%)	18.3 ± 4.7	(7.8–25.3)
Fat mass (kg)	13.9 ± 4.6	(5.6–20.5)
Non-bone lean mass		
Arms (kg)	8.9 ± 1.6	(6.4–11)
Legs (kg)	20.5 ± 4.2	(13.9–26.8)
Trunk (kg)	28.0 ± 4.1	(20–33.6)
Total (kg)	61.1 ± 10.0	(43.6–74.8)
Bone mineral density (g⸱cm^−2^)	1.4 ± 0.1	(1.2–1.6)
Bone mineral content		
Arms (kg)	0.5 ± 0.1	(0.4–0.6)
Legs (kg)	1.2 ± 0.2	(0.8–1.6)
Trunk (kg)	1.0 ± 0.2	(0.7–1.4)
Total (kg)	3.4 ± 0.6	(2.3–4.3)
Maximal isometric handgrip strength (kg)		
Dominant hand (kg)	56.6 ± 14.4	(35.5–77.5)
Non-dominant hand (kg)	54.6 ± 13.2	(34.1–77.5)
Total (kg)	111.1 ± 27.1	(70.8–155.0)
Difference (kg)	3.6 ± 4.4	(−3.4–10.4)
Bilateral strength asymmetry (%)	7.8 ± 5.0	(0.0–15.1)
Bench press strength		
Absolute (kg)	100 ± 24.2	(47–141.1)
Relative (kg⸱body mass^−1^)	1.3 ± 0.3	(0.7–1.7)
Glute bridge strength		
Absolute (kg)	198.9 ± 38.5	(128–243.7)
Relative (kg⸱body mass^−1^)	2.5 ± 0.5	(1.9–3.3)
Prone bench row strength		
Absolute (kg)	79.8 ± 12.9	(52.2–107.1)
Relative (kg⸱body mass^−1^)	1.0 ± 0.2	(0.8–1.3)

**Table 2:** Relationships between measures of body composition, training experience, and BJJ preferences.

	Belt Rank			Experience						Gi Preference						Fighting Style	
				BJJ			RT			Training			Competition				
	τ	BF₁₀		τ	BF₁₀		τ	BF₁₀		τ	BF₁₀		τ	BF₁₀		τ	BF₁₀
Height	0.27	0.7		0.16	0.5		0.14	0.4		−0.42	2.2		−0.57 *	5.3		−0.02	0.4
Body mass	0.16	0.5		−0.01	0.4		0.03	0.4		−0.43	2.4		−0.35	1.0		0.06	0.4
Body fat percentage	−0.53 *	6.5		−0.66 *	30.8		−0.22	0.6		−0.37	1.4		0.20	0.5		0.05	0.4
Fat mass	−0.31	0.9		−0.44 *	2.6		−0.05	0.4		−0.43	2.4		0.04	0.4		0.16	0.5
Non-bone lean mass																	
Arms	0.22	0.6		0.12	0.4		0.26	0.7		−0.20	0.5		−0.18	0.5		0.19	0.5
Legs	0.40	1.8		0.23	0.6		0.23	0.6		−0.27	0.7		−0.44	1.8		0.09	0.4
Trunk	0.40	1.8		0.20	0.5		0.26	0.7		−0.33	1.1		−0.53 *	3.6		−0.03	0.4
Total	0.40	1.8		0.23	0.6		0.26	0.7		−0.27	0.7		−0.44	1.8		0.09	0.4
Bone mineral density	0.28	0.8		0.23	0.6		0.13	0.4		−0.20	0.5		−0.39	1.3		−0.28	0.8
Bone mineral content																	
Arms	0.28	0.8		0.12	0.4		0.13	0.4		−0.20	0.5		−0.48	2.5		0.06	0.4
Legs	0.31	0.9		0.23	0.6		0.16	0.4		−0.20	0.5		−0.66 *	12.4		−0.06	0.4
Trunk	0.31	0.9		0.20	0.5		0.08	0.4		−0.40	1.8		−0.57 *	5.3		−0.25	0.7
Total	0.34	1.1		0.23	0.6		0.16	0.4		−0.30	0.9		−0.57 *	5.3		−0.13	0.4

* = Significant (*p* < 0.05) relationship.

**Table 3:** Relationships between measures of strength, training experience, and BJJ preferences.

	Belt Rank			Experience						Gi Preference						Fighting Style	
				BJJ			RT			Training			Competition				
	τ	BF₁₀		τ	BF₁₀		τ	BF₁₀		τ	BF₁₀		τ	BF₁₀		τ	BF₁₀
MIHS																	
Dominant	0.25	0.7		0.18	0.5		0.01	0.3		0.13	0.4		−0.35	1.0		0.16	0.5
Non-dominant	0.28	0.8		0.15	0.4		0.16	0.4		−0.20	0.5		−0.31	0.8		0.06	0.4
Total	0.22	0.6		0.12	0.4		0.01	0.3		0.03	0.4		−0.35	1.0		0.09	0.4
Difference	−0.16	0.5		0.01	0.4		0.03	0.4		0.30	0.9		0.09	0.4		0.25	0.7
Asymmetry	−0.25	0.7		−0.04	0.4		0.08	0.4		0.20	0.5		0.01	0.4		0.19	0.5
Bench press																	
Absolute	0.10	0.4		0.04	0.4		0.57 *	9.9		0.01	0.3		0.01	0.4		0.19	0.5
Relative	−0.04	0.4		−0.07	0.4		0.52 *	5.6		0.43	2.4		0.35	1.0		0.19	0.5
Glute bridge																	
Absolute	0.22	0.6		0.2	0.5		0.73 *	75.1		0.03	0.4		−0.39	1.3		0.19	0.5
Relative	−0.02	0.4		0.07	0.4		0.52 *	5.6		0.37	1.4		−0.09	0.4		0.31	1.0
Prone bench-pull																	
Absolute	0.19	0.5		0.07	0.4		0.44 *	2.6		0.17	0.5		−0.22	0.6		0.13	0.4
Relative	0.04	0.4		0.07	0.4		0.29	0.8		0.70 *	51.9		0.31	0.8		0.22	0.6

* = Significant (*p* < 0.05) relationship.

**Table 4:** Relationships between measures of barbell velocity, training experience, and BJJ preferences.

	Belt Rank			Experience						Gi Preference						Fighting Style	
				BJJ			RT			Training			Competition				
	τ	BF₁₀		τ	BF₁₀		τ	BF₁₀		τ	BF₁₀		τ	BF₁₀		τ	BF₁₀
Bench press velocity																	
Peak at 7 kg	−0.40	1.8		−0.39	1.7		0.23	0.6		−0.17	0.5		−0.22	0.6		0.38	1.5
Peak at 30%	−0.19	0.5		−0.23	0.6		−0.08	0.4		−0.40	1.8		−0.44	1.8		0.35	1.2
Peak at 40%	−0.22	0.6		−0.12	0.4		−0.44 *	2.6		−0.20	0.5		0.01	0.4		−0.03	0.4
Peak at 50%	−0.12	0.4		−0.04	0.4		−0.20	0.5		−0.10	0.4		0.24	0.6		0.40	1.7
Peak at 60%	−0.30	0.9		−0.30	0.9		−0.38	1.5		−0.32	1.0		−0.31	0.8		0.19	0.5
Average at 7 kg	−0.31	1.0		−0.31	0.9		0.28	0.8		−0.19	0.5		−0.31	0.8		0.41	2.0
Average at 30%	−0.09	0.4		−0.12	0.4		0.11	0.4		−0.29	0.8		−0.52 *	3.5		0.31	0.9
Average at 40%	−0.06	0.4		−0.08	0.4		−0.04	0.4		−0.17	0.5		−0.50	2.8		0.06	0.4
Average at 50%	−0.15	0.4		−0.18	0.5		0.08	0.4		−0.19	0.5		−0.58 *	5.8		0.30	0.9
Average at 60%	−0.16	0.5		−0.20	0.5		−0.05	0.4		−0.25	0.7		−0.67 *	14.0		0.32	1.0
Glute bridge velocity																	
Peak at 7 kg	−0.19	0.5		−0.18	0.5		0.36	1.4		−0.07	0.4		−0.26	0.7		0.16	0.5
Peak at 30%	−0.19	0.5		−0.23	0.6		0.29	0.8		−0.37	1.4		−0.26	0.7		−0.09	0.4
Peak at 40%	−0.16	0.5		−0.20	0.5		0.36	1.4		−0.10	0.4		−0.18	0.5		0.01	0.3
Peak at 50%	−0.34	1.2		−0.41	1.9		0.04	0.4		−0.05	0.4		0.01	0.4		−0.19	0.5
Peak at 60%	−0.36	1.3		−0.45 *	2.8		0.21	0.6		−0.10	0.4		−0.07	0.4		−0.08	0.4
Average at 7 kg	−0.19	0.5		−0.18	0.5		0.44*	2.6		−0.03	0.4		−0.35	1.0		0.22	0.6
Average at 30%	−0.22	0.6		−0.25	0.7		0.21	0.6		−0.36	1.3		−0.13	0.4		−0.11	0.4
Average at 40%	−0.25	0.7		−0.28	0.8		0.29	0.8		−0.03	0.4		−0.13	0.4		0.06	0.4
Average at 50%	−0.39	1.6		−0.46 *	3.1		0.04	0.4		−0.02	0.4		0.04	0.4		−0.17	0.5
Average at 60%	−0.33	1.0		−0.46 *	3.1		0.09	0.4		−0.08	0.4		0.02	0.4		−0.08	0.4
Prone bench row velocity																	
Peak at 7 kg	−0.28	0.8		−0.39	1.7		0.18	0.5		−0.23	0.6		0.04	0.4		0.38	1.5
Peak at 30%	−0.28	0.8		−0.34	1.1		0.13	0.4		−0.27	0.7		−0.18	0.5		0.31	1.0
Peak at 40%	−0.09	0.4		−0.19	0.5		0.41	1.9		−0.13	0.4		−0.18	0.5		0.21	0.5
Peak at 50%	−0.04	0.4		−0.20	0.5		0.10	0.4		−0.37	1.4		−0.35	1.0		0.13	0.4
Peak at 60%	0.31	0.9		0.20	0.5		0.47 *	3.3		0.01	0.3		−0.22	0.6		0.13	0.4
Average at 7 kg	−0.31	1.0		−0.39	1.7		0.20	0.5		−0.25	0.7		0.09	0.4		0.32	1.0
Average at 30%	−0.22	0.6		−0.31	0.9		0.10	0.4		−0.30	0.9		−0.26	0.7		0.28	0.8
Average at 40%	−0.19	0.5		−0.28	0.8		0.26	0.7		−0.33	1.1		−0.13	0.4		0.16	0.5
Average at 50%	−0.06	0.4		−0.22	0.6		0.07	0.4		−0.40	1.9		−0.26	0.7		0.14	0.4
Average at 60%	0.11	0.4		−0.03	0.4		0.17	0.5		−0.41	2.0		−0.20	0.5		0.13	0.4

* = Significant (*p* < 0.05) relationship.

## Data Availability

All data will be made available by the corresponding author upon request.
